# The effect of physiological levels of South African puff *adder (Bitis arietans)* snake venom on blood cells: an *in vitro* model

**DOI:** 10.1038/srep35988

**Published:** 2016-10-24

**Authors:** Morné A. Strydom, Janette Bester, Sthembile Mbotwe, Etheresia Pretorius

**Affiliations:** 1 Department of Physiology, University of Pretoria, South Africa

## Abstract

A significant burden of illness is caused globally by snakebites particularly by the puff adder, *Bitis arietans*. Presently there is no reliable and rapid method to confirm envenomation on blood chemistry; although coagulation parameters like prothrombin time, partial thromboplastin time, international normalized ratio and also serum electrolytes are tested. Here, we found that direct *in vitro* exposure of physiological relevant whole venom levels to human healthy blood (N = 32), caused significant physiological changes to platelet activity using a hematology analyzer, and measuring occlusion time, as well as lyses time, with the global thrombosis test (GTT). Disintegrated platelets were confirmed by scanning electron microscopy (SEM). We also confirmed the pathologic effects on erythrocytes (RBCs) (visible as eryptotic RBCs), by looking at both light microscopy and SEM. Thromboelastography showed that no clot formation in whole blood could be induced after addition of whole venom. We propose further clinical studies to investigate the use of light microscopy smears and hematology analyzer results immediately after envenomation, as a possible first-stage of clinical confirmation of envenomation.

A significant burden of illness is caused globally by snakebites, particularly in tropical and subtropical regions as South Asia, South-east Asia, and sub-Sahara Africa^1^. There is an annual worldwide estimate of more than 400 000 snake envenomation and 20 000 deaths^2^. In 2010, it was noted that the burden of human suffering caused by snake bite remains unrecognized, invisible, and unheard by the global public health community, and forgotten by development agencies and governments^3^. Six years later, nothing has changed.

The puff adder, *Bitis arietans*, is indigenous to sub-Saharan Africa and parts of the Middle East and is responsible for a significant amount of morbidity and mortality in these regions. Time to treatment is of great importance, especially with puff adder envenomation, because it is particularly cytotoxic, and may threaten limb or tissue loss at the bite site area. *B arietans* envenomation is characterized by tissue necrosis, hypotension, thrombocytopenia, spontaneous bleeding and severe coagulopathy may occur^4^,^5^,^6^,^7^,^8^. Isolated proteins from *B. arietans* venom have been shown to interact particularly with platelets.

- The Arg-Gly-Asy-containing peptide (Arietin), inhibited aggregation of platelets because it blocks aggregation through the interference of fibrinogen binding to fibrinogen receptors on the platelet surface^9^.
- Fibrinogenase (Ba100) from *B arietans* venom cleaves the Aα and Bβ chain of fibrinogen, rendering the molecule unable to polymerize into fibrin clots^10^.
- Botrocetin from *B. arietans* have also been found to agglutinate fixed or fresh platelets in the presence of von Willebrand factor (VWF) irrespective of the mammalian species^11^,^12^.
- Another cofactor from *B. arietans* venom, bitiscetin-2, also induces VWF-GPIb platelet interaction^13^.
- Recently, three novel peptides called baptides 1, 2 and 3 was identified, and up to now, these are the shortest peptides without disulfide bridges isolated from animal venom^11^. These peptides inhibit nicotinic acetylcholine receptors in a non-competitive way^11^; these nicotinic acetylcholine receptors are found on healthy platelets and is involved in aggregation^14^ and blockade of the receptor, mediates Ca^2+^ influx and considerably impairs platelet function.
- The protein, bitanarin, was also found to have high phospholipolytic activity (phospholipases A2 represent the most abundant family of snake venom proteins). Bitanarin directly affects the nicotinic acetylcholine receptors^15^. The authors found that the ability of proteins with high phospholipolytic activity (found in all types of venom with phospholipases A2 activity) interact with the nicotinic acetylcholine receptors and may be a general property of snake venom^15^. Bitanarin, in particular has structural similarity to PLA2s from Viperidae snake venoms, and possesses high calcium-dependent phospholipolytic activity.
- Other proteins in *B. arietans* venom were also found to affect platelet plug formation by interacting either with platelet integrins, membrane glycoprotein Ib (GPIb), or VWF^5^. Disintegrins purified from various snake venoms are strong inhibitors of platelet aggregation. Bitiscetin found in *B. arietans* venom, induce VWF-dependent platelet agglutination *in vitro*^5^.

At present, there is no reliable, rapid and validated method used to confirm envenomation on blood chemistry, although coagulation parameters like prothrombin time (PT) and partial thromboplastin time (PTT) and also international normalized ratio (INR) and serum electrolytes are usually tested. Generally, a syndromic approach is followed when envenomation is treated, and is focused only on signs and symptoms of envenomation. The clinical consensus in practice is to observe patient closely for signs and symptoms of envenomation, which usually manifest between 15 minutes and up to two hours after the snakebite, occurred. If none of the typical signs or symptoms of envenomation have been noted after two hours of clinical observation, the possibility of a dry bite (mechanical bite with no venom injected) is embraced by most clinicians^16^. Anti-venom dosage is based on the clinical response of the patient, where up to 6 to 8 vials of polyvalent anti-venom from the South African Vaccine Producers (SAVP) are administered as a typical baseline dosage in cases of puff adder envenomation. An up-titration approach is followed, until the envenomation parameters, based on the syndromic approach of envenomation, have subsided. However, in the case of puff adder bites, we strongly suggest that the time line of two hours may be detrimental to the health of the patient.

A great need for a fast, reliable and easily accessible clinical test is therefore identified to confirm snake bite envenomation. In conjunction with the typical cytotoxic effects inflicted by a significant puff adder bite (e.g. progressive soft tissue swelling), it is also of vital importance to determine if coagulation parameters, erythrocytes (RBCs) and particularly platelet activity are affected, as platelet activity may possibly be used as an acute phase reactant to confirm systemic envenomation. Despite the health burden of snakebites (and particularly puff adder bites) in South Africa, there is no standard guideline that support or suggest the use of platelet activity or whole blood smears as possible diagnostic tests. In this paper we investigate how puff adder (*B. arietans*) whole venom affects platelet, erythrocytes (RBCs), as well as fibrin fibre formation, using microscopy techniques. We also determine the effects of whole venom on platelets using a standard hematology analyzer; we measure platelet activity with the global thrombosis test (GTT) and finally, clot formation using thromboelastography (TEG).

## Methods

### Ethical statement

This study was approved by the Ethical Committee of the University of Pretoria (South Africa): ethics clearance number: 169/2016. A written form of informed consent was obtained from all healthy donors (available on request). The methods were carried out in accordance with the approved guidelines. Blood was collected and methods were carried out in accordance to the relevant guidelines of the ethics committee. We adhered strictly to the Declaration of Helsinki.

### Volunteer details and blood collection

Blood samples were obtained from 32 healthy individuals of ages ranging from 18 to 60. Blood was collected in two 4.5 mL citrate and EDTA tubes. Naïve, uncitrated blood was also collected in a syringe and this blood was dispensed immediately with and without venom into a global thrombosis test (GTT**^®^**) machine. A medical doctor did the blood collection and all handling of samples were performed under very strict aseptic conditions, in order to prevent contamination of samples.

### Snake venom used

Previous research suggested the use of 1 μg/ml for *B. arietans* added to platelet poor plasma^17^. In these experiments, lyophilized venom was reconstituted in calcium-free phosphate buffered saline (PBS, Sigma-Aldrich, Saint Louis, MO, USA) at a concentration of 50 mg/ml, aliquoted, and stored at −80 °C, until experiment.

The Transvaal Herpetology Association of South Africa donated whole venom from *B. arietans*. Whole venom was collected from 5 individual adult *B. arietans* specimens, from the same locality (Gauteng, South Africa). The venom was pooled to address minor inter-species variation of venom content. Aliquoted pooled venom was stored in its natural raw state at −80 °C. Although most venom samples used in research are lyophilized and centrifuged to eliminate cellular components and endogenous inhibitors before experimentation^18^,^19^, it was decided to investigate the holistic effects of raw venom on human blood for the purpose of this study. The rational for this approach is simulate the acute effects of *B. arietans* envenomation on blood ultrastructure and coagulation in humans.

Healthy whole blood was mixed at an exposure concentration of 0.4 μL per 4 mL whole blood for 10 minutes at room temperature, for all experiments except for the GTT where uncitrated blood was exposed for at least 30 seconds before initiation of the GTT experiment. This volume of venom is in line with typical physiological volumes induced into an individual after envenomation. The rationale for pre-exposure time of 10 minutes of whole venom to whole blood was to permit interaction time with the various blood components, so that the resultant fibrin coagulation kinetics and affects of the venom on the platelets and RBCs reflect either degraded or enhanced venom mediated change, prior to either biomechanical engagement of the TEG cup and pin, and other experimental procedures. This was in accordance with various recently published papers that investigated the effect of various snake venoms on plasma using particularly TEG analysis^17^,^20^,^21^.

### Hematology analyzer

Blood collected in EDTA tubes was used to test platelet numbers using a standard hematology analyzer (Samsung HC 10). This analyzer measures impedance by using the Coulter Principle for counting cells passing through an aperture. The volume distribution of the cells are typically displayed e.g. WBC, RBC, and PLT numbers.

### Global thrombosis test (GTT^®^)

Naïve, uncitrated blood was collected, and exposed for at least 30 seconds before it was dispensed into the GTT**^®^** machine. No activator or anticoagulant is used in the GTT, and it measures occlusion time (OT) and lyses time (LT) based on shear forces from ceramic balls. The experimental system therefore allows for minimum handling of naïve blood samples. During the experiment platelets in whole blood are exposed to high shear stress and become activated by a first ball bearing. There is a second ball bearing, and in the space between the two ball bearings, platelet aggregates are formed and thrombin is generated from the activated platelets^22^. The GTT**^®^** test measures platelet reactivity (occlusion time, OT), where an OT of less than 300 seconds indicates platelet hyper-reactivity, while a result between 300 and 500 indicates normal hemostatic/platelet activity and any value 900 and above is indicative of a bleeding risk^23^. It also shows the lyses time (LT), where an LT of less than 2000 seconds shows normal spontaneous thrombolytic activity and LT of 2000 to 4000 seconds shows a reduced thrombolytic activity^24^.

### Thromboelastography (TEG^®^)

TEG was used to study the viscoelastic clot properties (also known as coagulation kinetics) of the participants’ blood, before and after addition of whole venom. TEG is based on contact protein activation via interaction with a plastic cup and pin surface^17^. Blood in citrated tubes were used and standard TEG procedures were followed with addition of CaCl_2_ to activate the coagulation process as previously described^25^,^26^,^27^,^28^, where the final whole blood sample mixture volume of exposure concentration of 0.4 μL per 4 mL whole blood. From this exposure mixture, 340 μL and 20 μL of 200 mM CaCl_2_ was added into the TEG cup. Similarly, 340 μL of untreated whole blood and 20 μL of 200 mM CaCl_2_ were also added into the TEG cup. The TEG measures various elastic modulus-based parameters; see refs 17,26, 27, 28, 29 for detailed descriptions of the various parameters. All data were collected at 37 °C for 30 min.

### Light and scanning electron microscopy (LM and SEM) of whole blood and platelet poor plasma

#### Light microscopy

Blood smears (from blood with and without whole venom exposure) were made from citrated blood and stained with methylene blue and eosin, following a standard staining protocol.

#### Scanning electron microscopy

For SEM analysis, 10 μl of whole blood (with and without venom) were placed directly on a glass cover slip. We prepared both platelet rich plasma (PRP) and platelet poor plasma (PPP) by centrifuging whole blood (15 minutes for PRP and 30 minutes for PPP), with and without added whole puff adder venom. 10 μl of PRP was also placed directly on a glass cover slip.

To create extensive fibrin fibre networks; we added 5 μl thrombin to 10 μl of PPP (with and without exposure to venom). The South African National Blood Service (SANBS) supplied human thrombin, which was at a stock concentration of 20 U/ml and was made up in a PBS containing 0.2% human serum albumin. The WB, PRP smears and fibrin fibre network smears were fixed using 4% formaldehyde, dehydrated, dried, mounted and coated with carbon, according to previously described standard SEM preparation methods^30^. A Zeiss cross beam electron microscope was used to study the surface morphology of RBCs, platelets, and fibrin fibres. Due to the high quality of the SEM images, no processing was done except to add color using Adobe^®^Photoshop CS6^®^ version 13.0 × 64.

### Statistical analysis

Statistical analysis was done with StatsDirect and p-values were obtained using non-parametric Mann-Whitey analysis.

### Raw data storage

Raw data is stored on Onedrive that is an open access storage database (https://1drv.ms/f/s!AgoCOmY3bkKHgkFy7q1sVsxRv_2s) and on the corresponding author’s researchgate profile, https://www.researchgate.net/profile/Etheresia_Pretorius, as raw data. Also included are all our original images without added color and our laboratory’s step-by-step SOPs).

## Results

### Hematology analyzer results

We observed changes in platelet numbers, as measured by a standard hematology analyzer (see Table 1), Platelet numbers were significantly decreased for all individuals after venom exposure (P < 0.0001). This might be due to the direct cytotoxic effect of puff adder venom on platelets. Due to possible calcium-dependent phospholipolytic activity after exposure to the venom, platelet content is released, followed by platelet disintegration, and therefore the hematology analyzer cannot measure platelets as an intact entity anymore.

### Global Thrombosis Test (GTT) results

GTT determines only platelet activity, by measuring occlusion time (OT) and lyses time (LT). Results of occlusion time (OT) as well as lyses time (LT), as measured by the global thrombosis test (GTT) are shown in Table 1. An OT of less than 300 seconds indicates platelet hyper-reactivity and above 900 seconds shows possible bleeding risk. An LT of between 2000 and 4000 seconds indicates low thrombolytic activity and above 6000 seconds indicates a lack of thrombolytic activity in healthy individuals.

Non-parametric Mann-Whitney analysis of our GTT results showed that there is a significant difference between the LT before and after venom treatment (P < 0.0001). We statistically analyzed the OT values using the total sample, before and after treatment with venom, and we found no significant difference (P = 0.13). However, in a closer analysis, we saw that, in a group of healthy individuals after *in vitro* envenomation, OT was increased (a value above 300; Table 1: sample 1 to 13), while in the rest of the sample, the opposite was noted (decreased OT, less than 300). When we compared these 2 OT venom-added groups (group 1: sample 1 to 13 and group 2: sample 14 to 32) with each other, there was a significant difference (P < 0.0001).

### TEG results

The TEG measures clot formation and it has been used previously in various studies using various types of snake venom^17^,^20^,^21^, but whole blood analysis was not performed previously. During healthy clot formation, R-time is the clot reaction time measured in minutes, and it shows time of latency from start of test to initial fibrin formation. In our study no clot initiation could be induced using this method, and therefore, no R-time (i.e. clot initiation time) could be established. Results are not shown here.

### Scanning electron microscopy of RBCs, platelets and fibrin fibres

**Figure 1:**
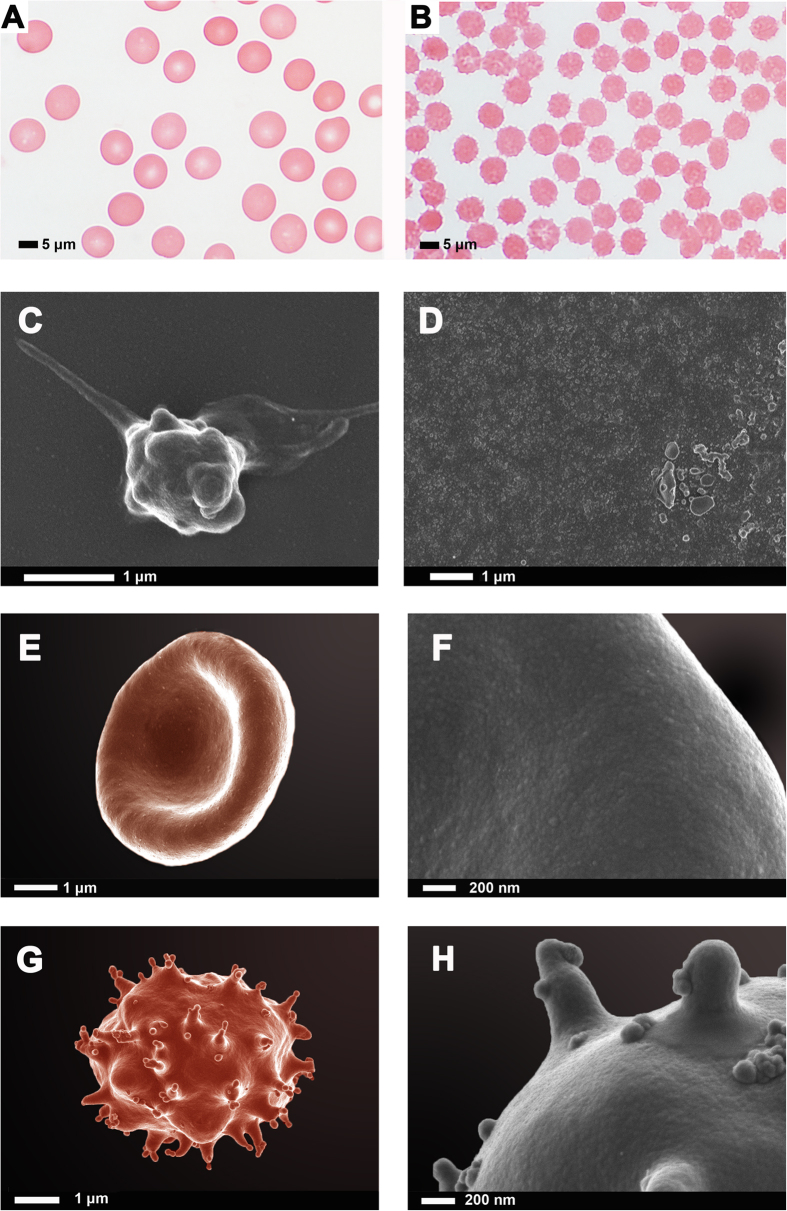
(**A**) A representative blood smear of a naïve blood smear; **(B)** after whole blood exposure to snake venom. (**C)** A typical platelet in a PRP smear. **(D)** No platelets recognizable in a PRP smear after exposure to snake venom. **(E)** A representative healthy erythrocyte. **(F)** Healthy erythrocyte membrane at high magnification. **(G)** An erythrocyte from the same individual, after 10 minutes exposure to puff adder venom. **(H)** High magnification of the venom-exposed erythrocyte membrane.

We noted pathologic effects on RBCs in whole blood and platelets in PRP, by looking at their ultrastructure using both light microscopy and SEM (Fig. 1). Figure 1A,B show representative light microscopy smears before and after venom exposure. SEM analysis showed RBC eryptosis after venom exposure and in PRP after venom exposure, only platelet remnants, with no individual platelets, were visible. Only matted granular sediments remained after venom exposure (Fig. 1C,H).

**Figure 2:**
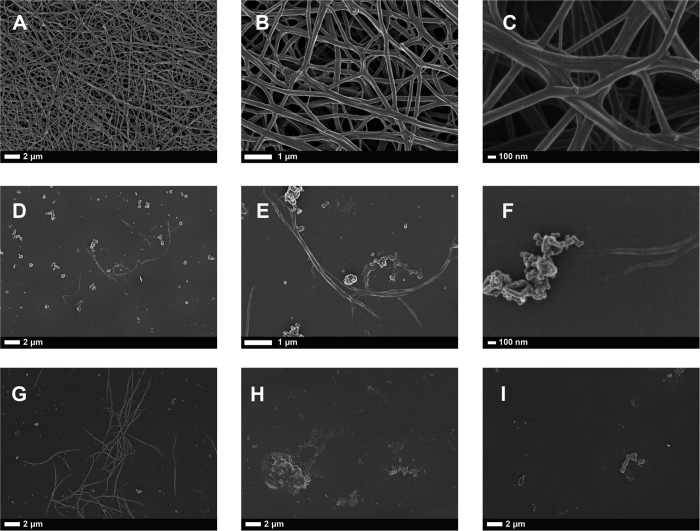
(**A to C**) Healthy fibrin fiber structure at various machine magnifications (10 000x; 35 000x and 100 000x); (**D to F)** the same individual as in (**A to C**), but with added venom; (**G to I)** micrographs from PPP with added venom, from other individuals to show the repeatability of results.

We also added thrombin to PPP (with and without added puff adder venom), to create extensive fibrin fibre networks. Thrombin activity is the last part in the coagulation pathway to create fibrin fibre nets from fibrinogen. In healthy fibrin clots, individual fibre fibers were noted, forming a clearly visible fibrin net. Figure 2A to C show typical fibre structures at 10000 x, 25000x and 100000x machine magnification. Figure 2D to F show micrographs of the same individual, at the same machine magnifications as Fig. 2A to C, where venom was added. Figure 2G to I show PPP with added venom from other individuals in our sample, to confirm repeatability of results (also see our raw data of all the PPP smears, stored, as noted in the materials and methods section). Where venom was added, very few sparsely formed fibers were noted, but mostly, thrombin could not succeed to activate venom-exposed PPP to form extensive fibrin fibre networks. Mostly, uncoagulated PPP proteins were found in the PPP with added thrombin, smears; with only a few fibres that could be detected.

## Discussion

*B arietans* venom is a known agent that causes coagulopathy, thrombocytopenia, and spontaneous bleeding in patients after envenomation. In this paper, our aim was to assess the effects of whole venom on blood cells and coagulation, to simulate as closely as possible, in the laboratory, the effects of clinical envenomization. Platelet counts were significantly reduced as seen with the hematology analyzer, and we suggest that this is due to platelet hyper-reactivity and disintegration. This was confirmed with PRP SEM analysis, where just a platelet-sediment remained in the presence of whole venom. As mentioned in the results, our OT GTT analysis of the whole sample, after addition of whole venom, did not show a significant difference between the naïve and venom-treated results. However, closer analysis indicated that there were two distinct patterns that were noted. There was a group of individuals where the OT was greatly increased (Table 1: sample 1 to 13), while in the rest of the sample, the opposite was noted.

There were therefore two scenarios:

1. Platelet-hyperactivity (OT less than 300) or
2. A bleeding risk (in this group there were 8/13 with an OT of 900 and the rest with an OT over 300; see Table 1). A bleeding risk is known to be indicative of puff adder envenomation. Most literature suggest that puff adder venom inhibit platelet aggregation, but there are proteins present that cause enhanced platelet agglutination, including the protein bitiscetin, that might be the cause of the noted platelet-hyperactivity^5^. These two scenarios in our healthy population, after *in vitro* envenomation, might be of importance for future clinical studies.

After snake venom exposure, the LT was increased for the whole sample, suggesting a low or lack of thrombolytic activity. We have noted in our SEM of PRP smears that platelets have disintegrated after venom exposure. We suggest here that because of this disintegration and possibly “blowing up” of the platelets after venom exposure, as the venom most probably causes a thrombin burst, that makes a primarily or lysed cell part plug. This plug would have no reason to lyse, as there would be no platelets gradually releasing their GPIIb/IIIa grip on the fibrin matrix.

TEG analysis confirmed that, with venom exposure no clots were initiated. We confirmed the TEG results with SEM analysis of fibrin fibres exposed to whole venom. We found that PPP exposed to venom and under the action of thrombin, does not from proper fibrin fibres, and suggest that the venom possibly impacts on the packaging-capability of the individual fibrin molecules. We have, over the past few years, published extensively on the ultrastructure of healthy fibrin fibre networks, and compared the structure to that of fibrin fibres of various inflammatory conditions^29^,^31^,^32^,^33^,^34^. Uncoagulable plasma proteins were mostly visible after addition of thrombin and only a few fibres formed. RBCs also showed eryptosis and this is an indication that whole puff adder venom impacts on the cellular structure and triggers eryptotic pathways.

These laboratory-simulated results may give important insights into more streamlined clinical interventions. From a point-of-care perspective, the first treatment will usually happen in primary healthcare hospitals, and at these facilities, most clinicians will have access to a complete blood count. A light microscopy blood smear may immediately confirm a changed RBC structure, as shown in Fig. 2B. Therefore, immediate analysis of platelet numbers will be an exceptionally easy and fast analysis. Platelet dysfunction can also easily be confirmed using a hematology analyzer, if present in a primary care facility. This should be investigated further in a clinical setting.

## Conclusion

In conclusion, bleeding may be an expected clinical sign in a snake bite patient, where not only blood cells, coagulation and fibrinolysis are involved, but also endothelial cells and blood flow have an enormous importance. The resulting pathophysiology is therefore enormously complex. Our *in vitro* results suggest that it might be important to further investigate the use of both light microscopy smears and results from a hematology analyzer as a possible screening mechanism if envenomation is suspected. The effectiveness of the suggested methods that we used in this *in vitro* laboratory study could also be investigated further in a clinical study or animal study. We conclude that a pro-active clinical approach that might include our suggested techniques, will not only save lives, but will also allow for informed management of anti-venom usage.

### Ethical approval disclosure

Ethical approval was granted at the University of Pretoria (HUMAN ETHICS COMMITTEE: FACULTY OF HEALTH SCIENCES): E Pretorius and MA Strydom (169/2016).

## Additional Information

**How to cite this article**: Strydom, M. A. *et al.* The effect of physiological levels of South African puff *adder (Bitis arietans)* snake venom on blood cells: an *in vitro* model. *Sci. Rep.* **6**, 35988; doi: 10.1038/srep35988 (2016).

## Figures and Tables

**Table 1:** Platelet counts before and after venom were added (hematology analyzer results) and occlusion time and lyses time results from GTT test.

NO.	G	AGE	NAÏVE PLT COUNT	PLT COUNT WITH VENOM	NAÏVE WB: OT	NAÏVE WB: LT	WB WITH VENOM: OT	WB WITH VENOM:LT
1	M	31	172	28	383.0	2429	*847.3*	6000
2	F	20	351	46	151.9	1966	*900.0*	3348
3	F	33	243	22	259.2	1682	*774.7*	4245
4	F	56	275	39	419.1	2963	*589.8*	2469
5	M	26	248	34	304.3	1443	*900.0*	4112
6	M	23	165	23	277.8	2052	*337.4*	6000
7	F	27	158	10	339.2	1543	*900.0*	3195
8	M	19	194	15	148.5	1403	*899.0*	6000
9	M	49	199	9	444.0	1958	*900.0*	6000
10	M	60	174	9	406.1	3595	*900.0*	4643
11	F	58	270	24	589.1	1327	*900.0*	6000
12	F	56	199	10	561.0	5454	*900.0*	6000
13	M	20	184	18	500.6	1505	*900.0*	6000
14	M	32	382	12	337.3	1694	179.9	2869
15	M	42	179	47	153.1	2079	53.4	6000
16	F	21	226	12	444.6	1999	107.3	6000
17	M	30	254	13	328.1	2648	51.0	6000
18	F	20	243	28	238.9	1858	38.4	6000
19	M	19	210	8	341.6	1384	56.0	6000
20	M	21	220	16	320.1	1873	102.9	5617
21	F	22	339	17	492.4	1174	51.1	5124
22	M	22	221	12	361.6	1526	58.1	5500
23	F	23	252	33	614.5	1874	67.1	4826
24	F	50	244	8	360.9	3205	45.0	6000
25	F	23	231	18	344.5	2600	26.5	6000
26	M	65	313	8	506.8	1805	49.2	5086
27	M	19	209	31	438.9	1528	48.0	4506
28	F	24	191	19	554.1	2635	39.0	6000
29	M	19	212	16	579.9	3012	54.2	6000
30	F	44	321	21	544.2	1275	48.9	6000
31	M	20	199	10	584.6	1850	212.6	6000
32	F	37	328	12	396.5	1715	45.0	6000
**MEDIAN**			**223.5 (±57.5)**	**16.5 (±10.8)**	**499.6 (±130)**	**1827.5 (±847.1)**	**49.1 (±384)**	**6000 (±1053.3)**
